# Advancing towards a "one-button" screening solution for Cryo-EM data acquisition with Smart EPU

**DOI:** 10.1063/4.0000878

**Published:** 2025-10-27

**Authors:** Edward Pryor, Holger Kohr, Julio Ortiz, Fanis Grollios

**Affiliations:** 1 Thermo Fisher Scientific, Hillsboro, OR, USA; 2 Thermo Fisher Scientific, Eindhoven, NL, Netherlands

## Abstract

For many years, the Cryo-EM community is expecting that software automation will achieve the ultimate goal of providing a Single Particle Analysis (SPA) data acquisition workflow that requires no setup from the user's side [1,2]. Achieving that will allow many more researchers, who are not necessarily application or microscope experts an optimal use of time in high throughput cryo-electron microscopes. This is particularly important in industrial set-ups or entry-level labs where efficiency and ease of use are key adoption barriers. Moreover, it has been identified that one of the main bottlenecks in Single Particle Acquisition (SPA) is screening automation [2]. Thermo Scientific Smart EPU with EPU Multigrid already provides a workflow that enables screening of multiple samples in highly automated way [3]. We are now expanding on that with the goal to make SPA screening fully unattended. With the use of EPU Multigrid, EPU Quality Monitor (EQM) and Embedded CryoSPARC Live, and a novel set of AI/DL algorithms, we can now demonstrate a workflow that allows users to screen a full cassette of samples on an autoloader system without the need to pass through any manual setup tasks.

To achieve that level of automation, many parts of the workflow need to be optimized. User workflows for screening often include the acquisition and subsequent analysis of small datasets from numerous grids in different conditions to find optimal ice thickness and particle behavior [2]. This task is quite tedious: Users need to load each grid, select grid squares with a variation in ice thickness, identify foil holes, select foil holes with a variation in ice thickness, curate initial selections and afterwards perform data analysis to find favorable conditions for data acquisition. Smart EPU introduces a set of innovative AI/DL plugins that automate all the above steps. Experimental results show that the algorithms perform to a comparable level to an experienced user. We also combine these innovations with a redesigned UI to invoke the setup with just one click. This screening solution will be connected to EQM and Embedded CryoSPARC Live, so data analysis also happens in real time.

With these developments in place, users will be able to load the samples, immediately start the screening process, and after a few hours get the results visualized in our data integration platform on their laptop.
